# Case Report: Clinical application of an *in vitro* prenylation assay in the diagnosis of an early-onset case of mevalonate kinase deficiency harbouring a novel *MVK* variant

**DOI:** 10.3389/fped.2025.1666672

**Published:** 2026-01-08

**Authors:** Alice Burleigh, Ovgu Kul Cinar, Paul Torpiano, Marcia A. Munoz, Charlotte Abell-King, Michael J. Rogers, Despina Eleftheriou, Paul A. Brogan

**Affiliations:** 1 Department of Infection, Immunity and Inflammation, University College London Great Ormond Street Hospital Institute of Child Health, London, United Kingdom; 2 Department of Rheumatology, Great Ormond Street Hospital for Children NHS Foundation Trust, London, United Kingdom; 3 Department of Immunology, Great Ormond Street Hospital for Children NHS Foundation Trust, London, United Kingdom; 4 Immune Biotherapies Program, Garvan Institute of Medical Research, Darlinghurst, NSW, Australia; 5 School of Clinical Medicine, UNSW Sydney, Sydney, NSW, Australia

**Keywords:** mevalonate kinase deficiency, protein prenylation, autoinflammation, genetics, clinical diagnostics, inflammasome, mevalonate pathway, IL-1β

## Abstract

Mevalonate kinase deficiency (MKD) is a systemic autoinflammatory disease caused by biallelic mutations in *MVK*. Individuals with MKD present with a recurrent fever syndrome, often including a skin rash, gastrointestinal symptoms and lymphadenopathy. The severity depends on the residual enzyme activity, which can be measured using an assay to confirm diagnoses in cases with non-confirmatory/novel *MVK* genotype. However, the assay is not widely available and utilises radioisotope, limiting its use in routine clinical care. More recently, the accumulation of unprenylated Rab GTPases in peripheral blood mononuclear cells, a downstream consequence of mevalonate kinase deficiency, has been described as an alternative diagnostic biomarker of MKD. We describe the utility of the Rab prenylation assay for the diagnostic workup of an infant with a novel *MVK* genotype presenting with fulminant autoinflammation. A seven-week-old girl, born to non-consanguineous White British parents, presented with features of haemophagocytic lymphohistiocytosis (HLH): fever, hepatosplenomegaly, bicytopenia, hyperferritinaemia, transaminitis, raised lactate dehydrogenase and C-reactive protein. She had a three-week history of fever and a generalised erythematous rash, with negative infectious work-up. Gene panel sequencing revealed biallelic trans *MVK* variants: *MVK*: c.151C > T (p.L51P), a previously described pathogenic variant; and *MVK*: c.1027C > T (p.L343P), a novel variant. A mevalonate kinase enzyme activity assay, requested via the reference laboratory in Amsterdam, confirmed 3% residual activity in the patient, consistent with MKD. However, this test result took 21 days to return, therefore a prenylation assay was performed in the meantime, revealing clear accumulation of Rab proteins in a blood sample from the patient, thus confirming pathogenicity of the variants and securing the diagnosis of MKD. The turnaround time of this assay was 2 days. We demonstrate the use of a protein prenylation assay in the diagnosis of a very early-onset case of MKD presenting with HLH, with a novel *MVK* genotype. This assay is quicker and simpler to set up in routine clinical care than measurement of mevalonate kinase activity, the current gold standard for MKD diagnosis. This case demonstrates the clinical utility of the prenylation assay for specific and timely MKD diagnosis and expands the genotypic spectrum of MKD.

## Introduction

Systemic autoinflammatory diseases (SAIDs) are a family of disorders defined by uncontrolled activation of the innate immune system, leading to systemic inflammatory episodes. Mevalonate kinase deficiency (MKD) is a monogenic SAID caused by biallelic mutations in *MVK*, which encodes a crucial metabolic enzyme of the mevalonate pathway. MKD encompasses a clinical spectrum; the milder form (also previously known as Hyper-IgD and periodic fever syndrome, HIDS) is an early-onset recurrent fever syndrome, often accompanied by skin rash, gastrointestinal symptoms, hepatosplenomegaly and lymphadenopathy (1). A more severe form, mevalonic aciduria, additionally includes failure to thrive, developmental delay, facial dysmorphia and neurological symptoms. The severity of clinical features depends on the level of residual mevalonate kinase (MK) enzyme activity, which is determined by the particular combination of biallelic *MVK* variants. Mevalonic aciduria (MA) is associated with lowest residual activity (<0.5% of normal), while higher residual activity (0.5%–20% of normal) is associated with the periodic fever phenotype (2–4). Measurement of MK enzyme activity (5) is therefore particularly helpful to confirm MKD diagnoses in cases with a non-confirmatory/novel *MVK* genotype. However, this test is not widely available. Currently, the only reference laboratory offering an MK enzyme assay is in Amsterdam, Netherlands; the turnaround time is 3–6 weeks, and the assay requires shipment of a fresh blood sample. It is unlikely that this test will ever become available in UK routine clinical care since the assay utilises radioisotope, which is heavily restricted in clinical laboratory settings.

The build-up of unprenylated Rab GTPase proteins has been described as an alternative diagnostic biomarker of MKD (6, 7). MK is an enzyme in the mevalonate pathway, required for the synthesis of isoprenoid lipids that are essential for the post-translational prenylation and hence membrane localisation of numerous proteins, including Rab GTPases (8). In MKD, insufficient MK activity reduces the metabolic flux through this pathway, resulting in lack of isoprenoid lipid biosynthesis and thus defective prenylation of small GTPases including Rab, Rho and Rac proteins (9–11). Loss of membrane-bound prenylated proteins, or the accumulation of dysfunctional unprenylated proteins in the cytosol, appears to enhance activation of the pyrin and/or NLRP3 inflammasome and cause hypersecretion of IL-1β (12, 13). Defective protein prenylation is also recapitulated in genetic mouse models of MKD (14) and THP-1 cell line models (15). We have optimised a biochemical assay that allows detection of unprenylated Rab proteins in cells, by enabling *in vitro* prenylation of Rab proteins in cell lysates using recombinant prenyl transferase and a biotinylated isoprenoid lipid substrate (16). Normally, unprenylated Rab GTPases are barely detectable in cell lysates. Hence, the intracellular accumulation of unprenylated Rab GTPases is highly indicative of MKD, distinguishing it from other IL-1β-mediated diseases such as Cryopyrin-associated periodic syndromes (CAPS) and Familial Mediterranean fever (FMF) (6, 7) that are caused by variants in *NLRP3* or *MEFV*, respectively. In theory, the prenylation assay could also help diagnose other genetic conditions in which disruption of the mevalonate pathway affects protein prenylation, for example in phosphomevalonate kinase deficiency caused by autosomal recessive mutations in the *PMVK* gene (17–19). Unlike the radioactive MK enzyme activity assay, the Rab prenylation assay does not require the use of radioactive reagents, and has a turnaround time of only two days, making it much more accessible in a routine clinical care setting.

Here, we describe the utility of the *in vitro* Rab prenylation assay in the diagnostic workup of an infant presenting with fulminant autoinflammation, with a non-confirmatory genotype suggesting possible MKD.

## Methods

### Ethical approval and consent

Full ethical approval for this study was given by the National Research Ethics Service, (ethics numbers 24/EM/0068 and 11/LO/0330)). Informed written consent/assent was obtained for the patient and her parents.

### Genetic testing

Patient genetic screening was performed through the UK National Health Service (NHS) using a large, targeted panel for primary immune deficiency (https://panelapp.genomicsengland.co.uk/panels/398/). For Sanger sequencing confirmation, PBMCs were extracted from patient and parental blood by Lymphoprep separation, and then DNA isolated using Gentra Puregene Blood Kit (Qiagen). Variants were confirmed by PCR and Sanger sequencing using the following primers for *MVK* exon 3 forward: 5′-CACTGGCTGTATCCTTGAACT-3′ and reverse: 5′-TCCTTGCACTCACCCAGAAA-3′ and *MVK* exon 10 forward: 5′-ACCATCTGAATGCCCTCGG-3′ and reverse: 5′-GGAGCTGAGACCTGTTTCCA-3′.

### MVK enzyme activity assay

Fresh patient blood was shipped to Amsterdam UMC laboratory, Amsterdam (https://www.amc.nl), where the standard MK enzyme activity assay was performed as previously described (5) in fresh patient lymphocyte lysates using the isotope R,S-[2-14C]-mevalonic acid and known Rf values of mevalonolactone, mevalonate 5-monophosphate and mevalonate 5-pyrophosphate.

### *In vitro* Rab prenylation assay

PBMCs were isolated from patient, parent, and healthy control blood by Lymphoprep separation, and stored in liquid nitrogen. The prenylation assay was performed as previously described (10, 14, 16). Briefly, cell lysates were generated from cryopreserved PBMCs by sonication in buffer (50 mM HEPES, 50 mM NaCl, 2 mM MgCl_2_, 100 μM GDP, protease inhibitor cocktail) and protein was quantified using a Pierce Rapid Gold BCA Protein Assay (Thermo Fisher Scientific). Unprenylated Rab proteins in cell lysates were prenylated in an *in vitro* reaction by incubating 10 mg protein from the lysates with 2 μM recombinant Rab geranylgeranyltransferase (GGTase II, Jena Bioscience), 2 μM Rab escort protein (Jena Bioscience), 0.5 μM biotin-tagged geranyl diphosphate (Jena Bioscience) and 2 mM DTT for 5 h at room temperature. Samples were then combined with Laemmli buffer supplemented with 10% beta-mercaptoethanol, boiled for 5 min at 95 °C, and separated by SDS-PAGE. Proteins were transferred to a PVDF membrane, washed with TBS-Tween (0.1%), blocked with 3% BSA, and incubated with streptavidin-HRP (Abcam) also in 3% BSA. Signal was detected using Amersham ECL Western Blotting Detection Reagent (Cytiva). Images were taken using a ChemiDoc Imager (Bio-Rad) and analysed using Image Lab software (Bio-Rad).

## Case presentation

**Figure 1:**
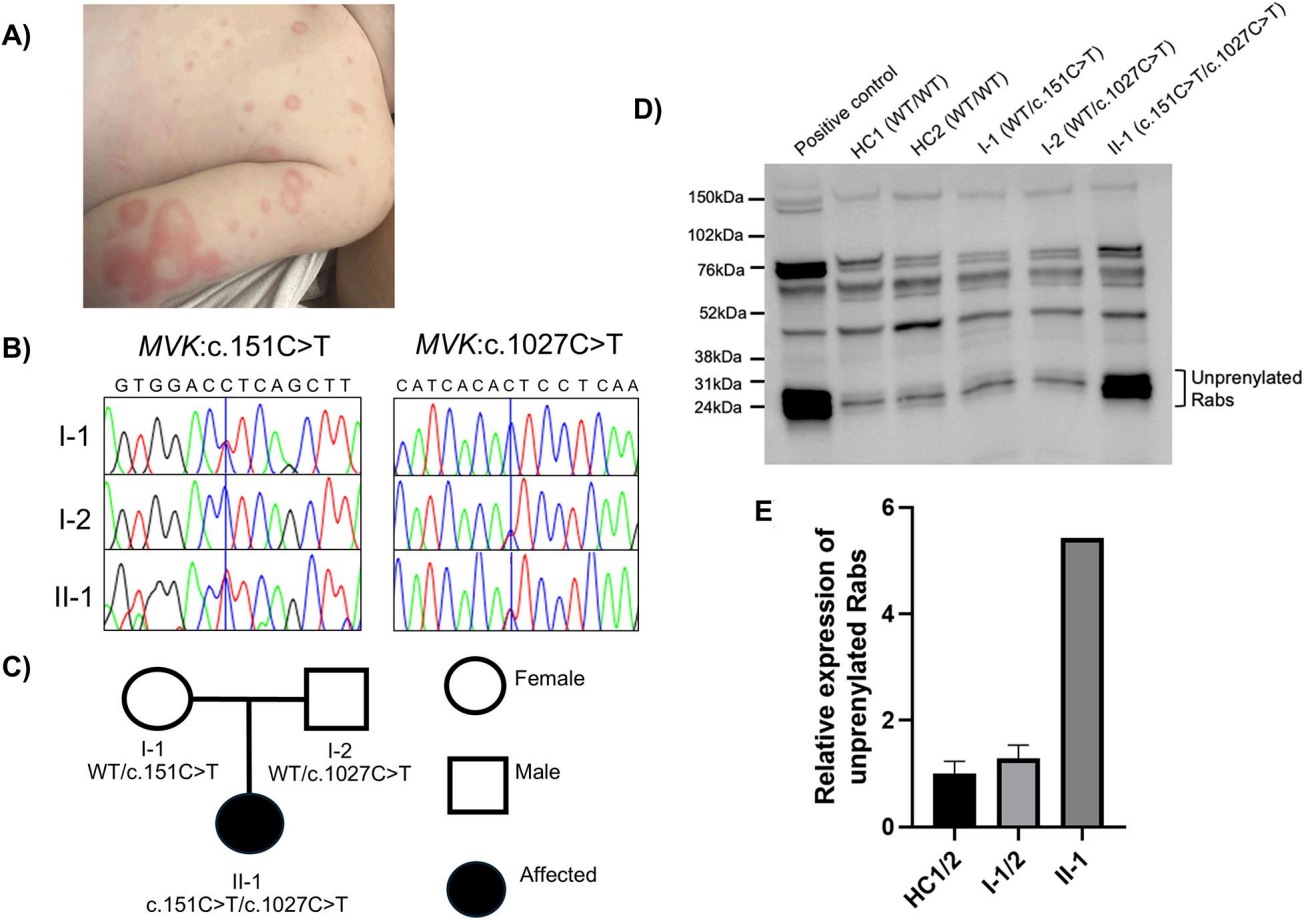
A very early onset case of mevalonate kinase deficiency (MKD) harbouring a novel *MVK* genotype. HC, healthy control; WT, wildtype. **(A)** Widespread erythematous, non-blanching rash at week 7 of age. **(B)** Sanger sequencing traces showing c.151C > T and c.1027C > T variants in the patient (II-1) and both parents (I-1 and I-2). **(C)** Pedigree and familial segregation of the *MVK* alleles with the affected individual marked in black and unaffected in white. **(D)** Following an *in vitro* prenylation reaction, cell lysate of PBMCs from the patient (II-1) shows clear accumulation of unprenylated Rab GTPases compared to two healthy controls and both heterozygous parents. The positive control (lane 1) shows THP-1 monocytic cells treated for 24 h with 0.5 mM simvastatin. **(E)** Densitometry analysis of the unprenylated Rab GTPase bands shows an increase in the presence of unprenylated Rab proteins in the patient PBMCs (II-1) compared with either healthy control (5.4-fold increase) or parent carrier (4.2-fold increase) PBMCs.

A seven-week-old girl, born at term via elective caesarean section to non-consanguineous white British parents from an uneventful IVF pregnancy, presented to the local hospital with features suggestive of haemophagocytic lymphohistiocytosis (HLH): fever, hepatosplenomegaly, bicytopenia (Haemoglobin (Hb):53 g/L (normal range:100–135 g/L), platelets:20 × 10*9/L (normal range:150–450 × 10*9/L), white cell count: 22.9 × 10*9 g/L (normal range: 6.00–18.00 × 10*9/L), lymphocytes:14.6 × 10*9 g/L (normal range:3.00–13.50 × 10*9 g/L), neutrophils:5.1 × 10*9 g/L (normal range:1.00–8.50 × 10*9 g/L), hyperferritinaemia (6,000 ug/L, normal range:9.5–75.7 ug/L), transaminitis (ALT: 690 U/L, normal range:12–47 U/L), raised lactate dehydrogenase (LDH) (911 U/L, normal range: 163–452 U/L) and a raised C-reactive protein (CRP) (60 mg/L, normal range: 0–5 mg/L). Parents reported illness onset at 4 weeks of life with fever and a generalised non-blanching erythematous rash (Figure 1A). During the first hospital admission with fever of unknown origin, she was extensively investigated for potential causes of sepsis and infectious screening was negative, including blood, urine, and cerebrospinal fluid (CSF) cultures and serum viral PCRs for Epstein–Barr virus (EBV), cytomegalovirus (CMV), adenovirus, enterovirus, varicella zoster virus (VZV) and parvovirus. CMV and toxoplasma gondii IgM were negative, IgGs were positive reflecting placental transmission. CSF viral PCRs for herpes simplex virus (HSV) 1 and 2, adenovirus, parechovirus, VZV and enterovirus were negative. Viral respiratory nasopharyngeal swab was negative. Chest x-ray and echocardiogram were normal. Maternal TORCH screening was negative. There was no other family history of note. The patient received 10 days of intravenous antibiotics (ceftriaxone and acyclovir) with complete resolution of rash, and she was discharged on oral acyclovir. Acyclovir was stopped following negative serum HSV PCRs.

At her second hospital admission with fever, poor feeding and a new skin rash that started as small erythematous papules and rapidly progressed to target lesions that coalesced on extremities, trunk, and face, she was found to be anaemic with Hb of 53 g/L and platelets were low at 20 × 10*9/L. She also was noted to have new hepatosplenomegaly, oedema, deranged liver functions and clotting with low fibrinogen (<1,5 g/L (normal: 1,7−4,0 g/L) requiring repeated doses of Vitamin K. She was covered with empirical IV antibiotics (ceftriaxone and gentamicin), and repeated blood and CSF cultures were negative. Blood film showed leucoerythroblasts, polychromasia, left shift and blast cells. Magnetic resonance imaging of the brain was normal. Ultrasound abdomen showed hepatosplenomegaly. Both kidneys looked normal and there was no proteinuria. Extended respiratory viral nasopharyngeal aspirate was negative. She remained transfusion dependent for red blood cells and platelets with no clear underlying diagnosis and was thus transferred to Great Ormond Street Hospital (GOSH) under the paediatric haematology team for diagnostic work-up and further management. On her transfer to GOSH, differential diagnoses included infections, malignancies, primary HLH, primary immunodeficiencies, and genetic causes of bone marrow suppression. Immunology, infectious diseases, haematology, and oncology teams were involved in the multidisciplinary diagnostic work-up. Perforin expression and CD107a granule release assays were normal (data not shown). Immunoglobulins (Ig)G and A were mildly raised [IgG: 9.81 g/L (2.10-7.70 g/L), IgA: 1.66 g/L (0.05-0.40 g/L)], and IgM was normal. Lymphocyte subsets with memory panel showed normal lymphocyte distribution and T cell types were also normal. Soluble CD25 was elevated at 15,613 pg/mL (normal range: 0–2,500 pg/mL); however, lymphocyte subset activation assay demonstrated normal CD25 expression patterns, indicating increased immune activation rather than an intrinsic defect in CD25 activation on T cells (data not shown).

Ultrasound of the abdomen showed a bulky liver with increased periportal echogenicity. There was no intrahepatic lesion with normal directional flow observed in the portal vein and hepatic veins. The gallbladder wall appeared thickened and oedematous with increased vascularity. Spleen was enlarged at 7.5 cm with the upper limits for age of 6 cm. There was moderate volume of anechoic free fluid, bilateral external iliac lymph nodes, largest in the left measuring 8 mm in short axis. A repeat chest x-ray was normal. Repeat echocardiogram confirmed normal intracardiac anatomy.

Bone marrow aspiration showed no phenotypic evidence of a haematological malignancy and no histopathological evidence of haemophagocytosis. Peripheral blood flow cytometry was normal. Metabolic investigations were normal apart from the urine organic acid test which showed presence of mevalonolactone. Blood electron microscopy demonstrated no ultrastructural indication of a storage disease. Extensive infectious work-up at GOSH showed a positive CMV IgM, positive CMV IgG and a positive serum CMV PCR with a viral load of 146,177 copies/mL whole blood. Other serum viral PCRs were all negative, including parvovirus, HIV, HHV-6, EBV, HSV 1 and 2, parechovirus, VZV, enterovirus and hepatitis A and C. Leishmania serology and culture on the bone marrow aspirate were negative. Although positive CMV PCR and CMV IgM were explained with postnatally acquired infection, the decision was to start treatment with an antiviral medication (valganciclovir) since CMV can be a potential trigger of an inflammatory process and HLH. Ophthalmology assessment for CMV retinitis and audiology test were both normal. There were no clinical signs of congenital CMV infection. After initiating antiviral treatment, CMV viral load started to reduce with improvement in blood counts (Hb and platelets) allowing her to be transfusion-independent and HLH parameters including ferritin, liver enzymes, fibrinogen and LDH normalised. Patient was discharged with pending genetic testing and oral valganciclovir treatment was stopped after a two-week course. Despite resolution of clinical symptoms and stability of blood counts, CRP remained mildly elevated around 25 mg/L (normal:0–20 mg/L).

Due to very early presentation with HLH features, the NHS England genetic panel for “Primary immunodeficiency disorders” was requested. This revealed biallelic *MVK* variants: *MVK*: NM_000431: c.151C > T (p.L51P), a rare variant previously observed in MKD (20, 21); and *MVK*: NM_000431: 1027C > T (p.L343P), a novel variant. The variants were inherited biparentally (in trans), as confirmed by parental Sanger sequencing (Figures 1B,C). At this stage, the patient was initiated on treatment with anti-interleukin 1 (IL1), anakinra administered as daily subcutaneous injections at a dose of 3 mg/kg/day. This led to the complete resolution of symptoms, normalisation of blood counts, including an increase in Hb from 86 g/L to 101 g/L (normal:100–135 g/L), a rapid reduction in CRP levels from 40 mg/L to 5 mg/L over 14 days, and normalisation of ferritin from 1,190 ug/L to 46.7 ug/L (reference range 9.5–75.7 ug/L). At the age of 9 months, she was converted from anakinra to canakinumab (4 mg/kg monthly) and remains well in both clinical and serological remission on this treatment at the time of writing, now 17 months of age.

To investigate the pathogenicity of the patient's variants, an MK enzyme activity assay (5) was requested via a reference laboratory in Amsterdam, since the test is not currently available in the UK. To the best of our knowledge, this is the only laboratory offering this test in Europe. After 21 days the results were returned, confirming 3% residual enzyme activity [level: 6 pmol/(min.mg protein), reference range: 125–395] in the patient, consistent with MKD.

The enzyme activity test result took 21 days to return, therefore an *in vitro* Rab prenylation assay (6) was performed in the meantime. This revealed a clear accumulation of unprenylated Rab GTPases in the patient PBMCs, compared with both healthy controls (5.4-fold increase) and carrier parents (4.2-fold increase) (Figures 1D,E). This result was consistent with previous observations for MKD patients (6, 7), confirming the pathogenicity of both variants *MVK*:c.151C > T (p.L51P) and *MVK*:1027C > T (p.L343P), and securing the diagnosis of MKD. The turnaround time of this assay was two days.

## Discussion and conclusions

We describe a case of early-onset MKD harbouring compound heterozygous *MVK* variants, one of which was previously described in a single symptomatic case (p.L51P), and the other novel (p.L343P). Our patient first presented at 4 weeks of life with fever and skin rash and then developed features of HLH at week 7, remained transfusion-dependent which led to her transfer to a quaternary centre and extensive investigations including genetic testing. To verify the pathogenicity of the two *MVK* variants, two functional assays were performed on PBMC samples—analysis of residual MK enzyme activity and accumulation of unprenylated Rab GTPase proteins. The results of both tests were consistent with MKD, confirming the pathogenicity of both variants; however, the Rab prenylation assay had a turnaround time of 2 days when performed in-house, compared with 21 days for the outsourced MK enzyme activity assay.

The development of clinically relevant functional tests to confirm genetic diagnoses has been relatively slow in comparison to advances in molecular genetics. This is particularly pertinent in the field of SAIDs, where many new diseases continue to be described, outpacing routine clinical laboratory functional assay development. Although MKD is well described and common pathogenic *MVK* mutations such as p.V377I are well characterised and highly prevalent in MKD patients [128/144 (86.0%) harbouring at least one copy in the largest cohort described (1)], some patients still present with compelling clinical phenotypes but non-confirmatory genotypes, mandating confirmation by measurement of residual MK enzyme activity. Despite this, availability of the MK enzyme activity assay is extremely limited, costly (when shipping costs of fresh samples are included), and time-consuming (3–6 weeks turnaround time). Simpler, cheaper and more rapid assays to confirm MKD, such as the *in vitro* Rab prenylation assay (6, 7, 10), are therefore needed.

Our case illustrates this point well, since she harboured one copy of an entirely novel *MVK* variant (p.L343P) combined with another known pathogenic variant (p.L51P), inherited biparentally. Our patient experienced severe gastrointestinal involvement with slow weight gain and vomiting since the start of her symptoms, and continuous rather than “periodic” autoinflammation, possibly explained by the relatively severe combination of *MVK* alleles. Audiological and ophthalmological evaluations of the patient were within normal limits, and neurodevelopmental milestones were appropriate for her age. There were no dysmorphic features suggestive of mevalonic aciduria, hence the patient was discharged from the care of the metabolic team. Ter Haar et al. found that absence of the p.V377I genotype had significant correlation with continuous course of disease, severe gastrointestinal involvement, and musculoskeletal involvement. Our patient's autoinflammatory phenotype suggested that neither the p.L51P or p.L343P variants have a severe enough impact on the protein structure to cause mevalonic aciduria (MA), consistent with both variants being outside “hotspot” regions of the enzyme associated with MA (residues 8–35 and 234–338) (22). However, the patient did show features consistent with HLH, which is a severe and unusual feature in MKD (23–25). Our patient did not have any primary HLH or primary immunodeficiency-related gene mutations, and we conclude that HLH in this instance was driven by MKD which can be associated with HLH with or without infectious triggers.

As we demonstrate in this case report, the MK enzyme activity assay and the Rab prenylation assay are both helpful in the diagnosis of MKD. Whilst reduced MK enzyme activity is diagnostic of MKD, the prenylation assay may theoretically detect insufficiency in other stages of the same metabolic pathway, for example in cases of *PMVK* deficiency (17–19). Whilst the gold standard MK enzyme activity has many strengths, there are also many practical limitations (Table 1). Most importantly, the MK enzyme activity assay is rather impractical for routine care, since it requires shipping of fresh sample to Amsterdam with a turnaround time of several weeks. It is unlikely that this assay will ever become routinely available in the UK since the use of radioisotopes is heavily restricted in routine clinical laboratory settings. In severe and life-threatening presentations with HLH features and persistent autoinflammation, as observed in our case, starting treatment promptly before the MK enzyme activity result becomes available may be critical in controlling inflammation and preventing devastating outcomes, particularly when MKD is the highly suspected diagnosis.

**Table 1:** Strengths and limitations of mevalonate kinase enzyme and Rab prenylation assays for routine diagnostic workup of suspected mevalonate kinase deficiency (MKD).

Assay	Strengths	Limitations
MK enzyme activity	Well-established/validated gold standard Directly measures activity of mutated MK protein in MKD Correlates well with clinical phenotypes (MA < 0.5% activity; MKD 0.5–20% activity)	Not available in the UK; only limited availability in Europe Turnaround time 3–6 weeks Requires international shipping of fresh sample Requires use of carbon-14 radioisotope, making it challenging/unlikely to be set up in routine clinical laboratories Requires use of a radioactivity measurement instrument, which are becoming less widely used in research and clinical labs Would require methodological adaptation to detect similar phenotypes caused by mutations in genes in the same pathway (e.g., *PMVK)*
Detection of unprenylated Rab proteins	Measures prenylation: a process that adds a lipid to a protein, impairment of which results in pyrin and NLRP3 inflammasome dysregulation in MKD 2-day turnaround time Does not require the use of radioisotope so easier to set up in routine clinical laboratories Requires only western blotting equipment Will detect similar phenotypes caused by mutations in alternative genes in the same pathway (e.g., *PMVK*)	Not yet well-established; limited understanding of true diagnostic utility (sensitivity/specificity) for MKD and similar phenotypes No standardised reference ranges yet available, hence internal controls required to be run per assay

MK, mevalonate kinase; PMVK, phosphomevalonate kinase; MA, mevalonic aciduria.

Another alternative assay used in the workup of MKD is the detection of mevalonic acid in urine samples. Although this may be of great importance to support a diagnosis, methodology influences the sensitivity and specificity of this test, and the timing of the assay in relation to disease activity is important. Urinary mevalonic acid levels are highly informative during symptomatic periods of MKD, with high sensitivity and specificity (26). This test, especially when using advanced techniques such as gas chromatography and mass spectrometry (which was the method used in this case) can provide a more specific indication of MKD. Overall, however, this test is a basic screening tool when genetic testing is unavailable, and often reported in a qualitative rather than a quantitative fashion, as in our case. Recent European guidelines emphasise that even when urinary mevalonic acid excretion is positive, analysis of the *MVK* gene remains essential to confirm the diagnosis of MKD (26).

Alternatively, the *in vitro* Rab prenylation assay does not rely on symptomatic periods to provide a positive result in MKD patients, and is simple to establish, requiring only western blotting equipment. The assay has a short turnaround time of two days and does not require the use of radioisotope. The assay can be performed using fresh or cryopreserved PBMCs, again making it much more accessible for use in routine clinical care. Although the extent of the protein prenylation defect appears to correlate with residual MK enzyme activity (7), analysis of a larger cohort is required to further define this relationship. The prenylation assay cannot, therefore, be used to predict the severity of disease. It can, however, be utilised in the diagnostic workup of cases when the MK enzyme activity assay is inaccessible or will delay the treatment of urgent cases with novel or non-confirmatory genotypes. The strengths and limitations of these assays for the diagnostic work-up are summarised in Table 1.

The rationale for the *in vitro* prenylation assay is that, in MKD, insufficient synthesis of isoprenoid lipids (due to the partial loss-of-function mutations in *MVK*), prevents the normal prenylation of a multitude of proteins, including numerous Rho/Rac/Rap1-family and Rab-family small GTPases. These unprenylated proteins can be detected by western blotting for unprenylated Rap1a, or by *in vitro* labelling using a prenylation assay with recombinant geranylgeranyltransferase I (GGTase I) or GGTase II (Rab GGTase (16). Although defective prenylation of specific GTPases such as Rac may play a role in triggering the inflammatory cascade in MKD [reviewed in (13)], we have previously shown that the detection of GTPases modified by GGTase I (such as Rac), or western blotting for unprenylated Rap1a, lacks sensitivity. In comparison, the *in vitro* prenylation assay with GGTase II to detect unprenylated Rab GTPases is at least 10-fold more sensitive, since PBMC have an abundance of different Rab GTPases that can be prenylated *in vitro* by GGTase II (16). Hence, unprenylated Rab proteins are the most sensitive surrogate marker of defective protein prenylation in general.

We demonstrate with this case the clinical utility of the *in vitro* Rab prenylation assay for specific and timely diagnosis of MKD and we expand the genotypic spectrum of MKD. We suggest that the prenylation assay has practical advantages over the traditional MK enzyme assay and will be increasingly relevant for the diagnostic workup of MKD and related genotypes as summarised in Table 1. Larger scale validation, with consideration of genotype-phenotype correlation matched with patient MK enzyme activity, will further expand our understanding of the strengths and limitations of this assay in routine practice.

## Data Availability

The original contributions presented in the study are included in the article/Supplementary Material, further inquiries can be directed to the corresponding author/s.
